# Unusual Iliopsoas Abscess due to *Salmonella typhi*

**DOI:** 10.4269/ajtmh.23-0833

**Published:** 2024-04-02

**Authors:** Harpreet Singh, Deba Prasad Dhibhar, Suresh Selvam, Vishal Kumar, Vikas Suri, Ashish Bhalla

**Affiliations:** ^1^ Division of Clinical Infectious Diseases, Department of Internal Medicine, Post Graduate Institute of Medical Education and Research, Chandigarh, India;; ^2^ Department of Orthopaedics, Post Graduate Institute of Medical Education and Research, Chandigarh, India

**Figure 1.**
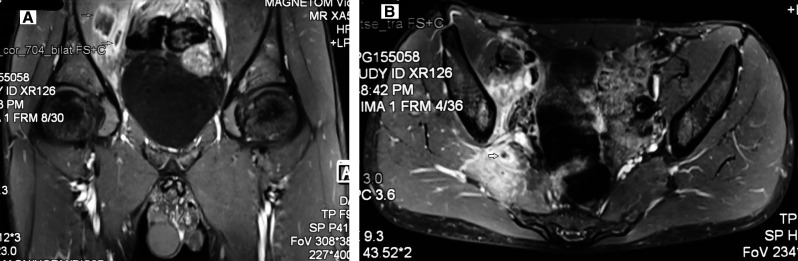
(**A**) Magnetic resonance imaging of the bilateral hip joints showing collection in the right iliopsoas muscle with surrounding edema (arrows = coronal section). (**B**) Magnetic resonance imaging of the bilateral hip joints showing collection in the iliopsoas muscle with surrounding edema (arrows = transverse section).

A 16-years old male was admitted with intermittent fever up to 103°F with chills and rigors initially for 4 days followed by intermittent spikes every 3rd or 4th day for total duration of 40 days. It was also associated with right buttock pain of pricking type with moderate intensity that increased on movement and was relieved with analgesics and rest; progressing to severe intensity with local redness, swelling over the right hip and inability to walk begining 2 days before admission. There was no recent history of gastroenteritis. Clinical examination revealed stable vitals with redness and swelling over the right iliac region with decreased range of movement at right hip joint and positive Faber test on right side with normal examination of the other joints. Laboratory investigations revealed hemoglobin of 13.2 g/dL, total leukocyte count of 13,300/cm^3^, platelet count of 265 × 10^9^/L with normal liver function tests and blood cultures. Magnetic resonance imaging of bilateral hips showed right side sacroiliitis with collections in right iliopsoas and piriformis muscle with size of 2.5 × 4.1 × 2 cm and surrounding edema (Figure 1A and B). His IgM *Brucella* serology, HLA-B27, and serum Widal were negative. Fine needle aspiration from the collection showed pus with growth of *Salmonella typhi* with sensitivity to ceftriaxone, azithromycin, and negative Genexpert for *Mycobacterium tuberculosis*. He was started on injection ceftriaxone 2 g intravenously twice a day for 4 days, which resulted in resolution of fever and later was changed to oral azithromycin 1 g daily for 5 days and oral cefixime 200 mg twice daily for 4 weeks. Screening for HIV was negative by serology. His hospital blood sugar recordings were normal. Screening for hemoglobinopathy (thalassemia and sickle cell disease) by high performance liquid chromatography was also normal. At 6-week follow-up, he is afebrile, pain has subsided, and range of movement at the right hip joint has improved.

Typhoid or enteric fever is caused by species of *Salmonella enterica* serovar typhi and by *Salmonella* paratyphi A in a smaller number of cases; it is transmitted through the fecal-oral route,^1^ and is prevalent in developing nations such as India, and can have various extaintestinal manifestations including focal abscess formation at different sites including spleen, subcutaneous soft tissues, and musculoskeletal involvement.^2^ Spread of these infections from primary gastrointestinal focus to distant organs or tissue occurs due to lymphohematogenous spread.^3^ Psoas abscess due to *Salmonella typhi* is an uncommon or rare presentation.^4^ In a study of 100 consecutive cases of focal *Salmonella* infection in 2006, 15% had focal abscess, and none had psoas abscess.^5^ In another study of 120 patients of bacteraemia due to *Salmonella* over a period of 10 years; 1.7% had psoas abscess.^6^ Various risk factors reported in the literature for focal *Salmonella* infection include poorly controlled diabetes mellitus, sickle cell anemia, and hemoglobinopathies.^7^ The likely manner of spread in our case was involvement of the right sacroiliac joint and then contiguous spread to the iliopsoas muscle. There are no definite guidelines for surgical versus medical management and duration of antibiotics in these types of focal *Salmonella* infections.^4^^,^^7^ We gave the patient a longer duration (4 weeks) of guided antibiotics in view of the focal muscle infection.
